# Aptamer-antibody hybrid ELONA that uses hybridization chain reaction to detect a urinary biomarker EN2 for bladder and prostate cancer

**DOI:** 10.1038/s41598-022-15556-1

**Published:** 2022-07-07

**Authors:** Eunseon Kim, Minji Kang, Changill Ban

**Affiliations:** Department of Chemistry, Pohang University of Science and Technology, 77, Cheongam-Ro, Nam-Gu, Pohang, Gyeongbuk 790-784 Republic of Korea

**Keywords:** Diagnostic markers, Sensors, DNA

## Abstract

We report an EN2-specific (*K*_d_ = 8.26 nM) aptamer, and a sensitive and specific enzyme-linked oligonucleotide assay (ELONA) for rapid and sensitive colorimetric detection of bladder and prostate cancer biomarker EN2 in urine. The assay relies on an aptamer-mediated hybridization chain reaction (HCR) to generate DNA nanostructures that bind to EN2 and simultaneously amplify signals. The assay can be performed within 2.5 h, and has a limit of detection of 0.34 nM in buffer and 2.69 nM in artificial urine. Moreover, this assay showed high specificity as it did not detect other urinary proteins, including biomarkers of other cancers. The proposed ELONA is inexpensive, highly reproducible, and has great chemical stability, so it may enable development of a simple, sensitive and accurate diagnostic tool to detect bladder and prostate cancers early.

## Introduction

Engrailed-2 (EN2) is an important marker for a wide variety of tumors^1–4^. It is a transcriptional factor that has important functions in embryonic development and oncogenesis^5,6^. It is excreted in patient urines of bladder and prostate cancer at levels that depend on the severity of the cancer^2,3,7^. Urine can be collected non-invasively, so assay of urine for the presence of EN2 may enable detection of these cancers at an earlier stage as a routine diagnosis.

Enzyme-linked immunosorbent assay (ELISA) is a simple test that requires little sophisticated equipment^8–10^. However, it uses special antibodies as molecular recognition elements for diagnosis. Recent modified ELISA uses aptamers because they have advantages of low cost, high stability, little batch-to-batch variability, and easy of synthesis and modification; the modified assay is called enzyme-linked oligonucleotide assay (ELONA), and also aptamer-linked immunosorbent assay (ALISA) or enzyme-linked apta-sorbent assay (ELASA)^11–14^.

Herein, we used systematic evolution of ligands by exponential enrichment (SELEX) to discover a single-stranded DNA (ssDNA) aptamer that has a great affinity toward EN2. The selected aptamer was applied to ELONA as detection agent by pairing with an EN2 monoclonal antibody and, at the same time, to hybridization chain reaction (HCR) for signal amplification; this combination enabled aptamer-mediated sensitive and specific detection of EN2. HCR is an isothermal and enzyme-free nucleic acid amplification technique that is appropriate for use in biosensors, because the reaction process can be completed without instruments such as thermal cyclers to control the variation in temperature^15,16^. Therefore, we combined HCR with ELONA to enable detection of EN2 by using only a conventional plate reader. The developed HCR-based aptamer-antibody hybrid ELONA could detect EN2 in buffer and artificial urine condition with high reproducibility, accuracy, and stability, and therefore may represent a powerful strategy to diagnose bladder and prostate cancer.

## Results

**Figure 1:**
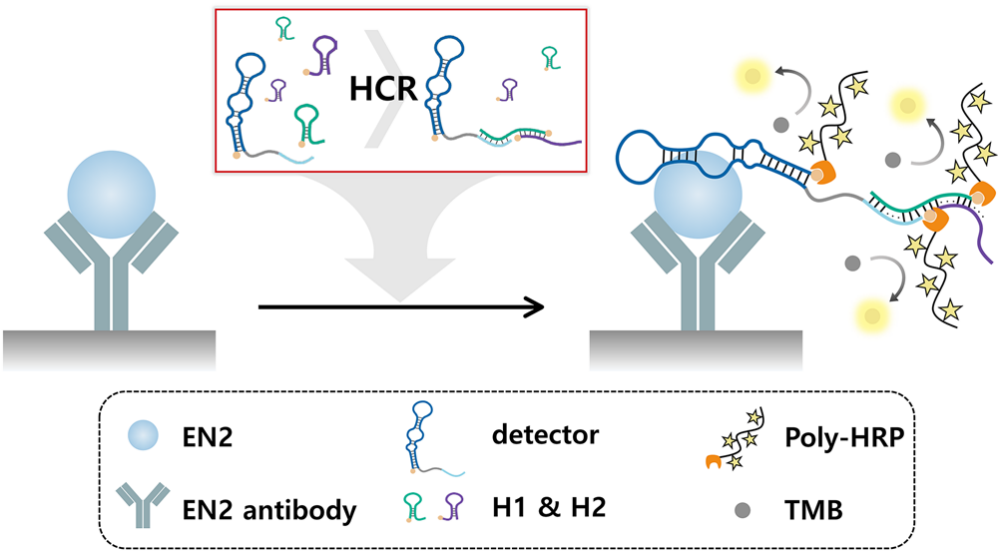
Schematic illustration of the HCR-based aptamer-antibody hybrid ELONA for EN2.

We designed an HCR-based aptamer-antibody hybrid ELONA system to detect EN2 (Fig. 1). The detector was fabricated by connecting EN2 binding aptamer (EBA) to a trigger sequence that can trigger HCR, and two auxiliary probes H1 and H2 that have hairpin structures were designed. The front part of the 5’ end of H1 complements the front part of the 5’ end of H2. The rear part of the 3’ end of H2 can hybridize with the rear part of the 3’ end of H1. Therefore, a series of hybridization events can lead to a long-range DNA nanostructure (composed of alternating H1 and H2) in the presence of the detector. When the target protein is present, it is captured by the antibody coated on the plate, and the detector can link the HCR product to the target by exploiting the interaction between EN2 and EBA of the detector. Thus, even a single molecule of the target can cause the association of the long nanostructure, and yield amplified colorimetric signals. In the absence of target protein, although the added H1 and H2 can self-assemble and form DNA nanostructure in solution, the DNA nanostructure cannot connect to the target. Also, poly-HRP was added to reduce the HRP binding time, and thereby reduce the total reaction time^17^.

### Development of ssDNA aptamer specific to EN2

**Figure 2:**
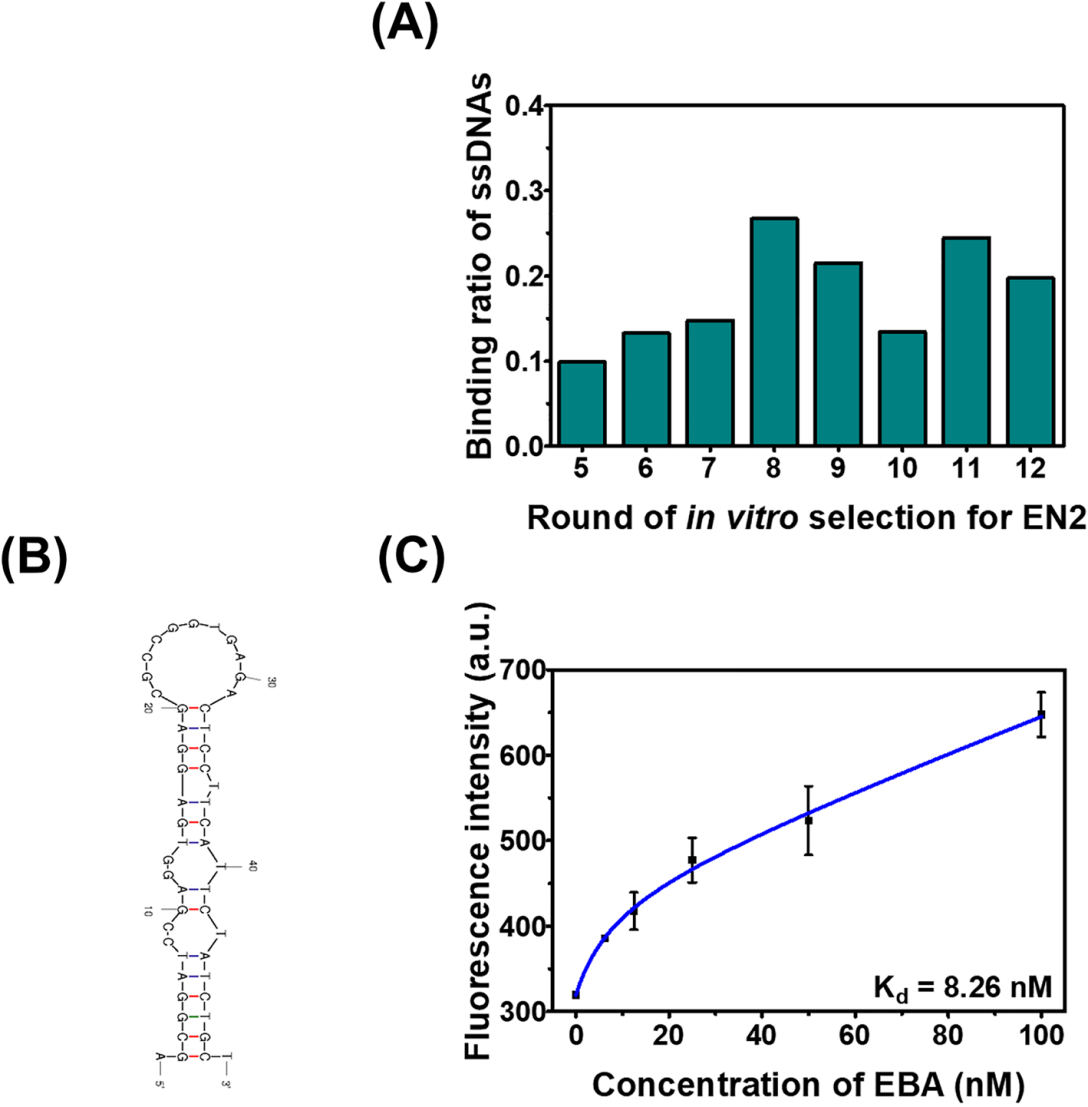
Development and characterization of EBA. (**A**) Binding ratio of ssDNA libraries to EN2 in each round. In order to obtain an aptamer with high affinity, the reaction time of each round was gradually reduced. The binding ratio increased up to round 8 and was almost maintained even under harsher binding conditions except for round 10. The PCR product of round 12 was used for sequencing, and the obtained H90 was trimmed with EBA. (**B**) Secondary structure of EBA in Mfold (http://www.unafold.org/mfold/applications/dna-folding-form.php). (**C**) Determination of the *K*_d_ value for EBA. A one site-total and nonspecific binding model was fit to the results of the fluorescent assay, and EBA showed the affinity value of 8.26 nM. Bars: ± s.d., n = 3.

N-terminal His-tagged recombinant EN2 protein was cloned and purified using a bacterial expression system and immobilized metal affinity chromatography (Fig. S1). The result of SDS-PAGE indicated the purity of the recombinant EN2 to be > 90% (Fig. S1C). The biological activity of the recombinant EN2 was confirmed by its affinity for a double-stranded DNA motif (5’-TAAT-3’) to which EN2 binds to regulate transcription (Fig. S2)^18–20^. Next, DNA aptamers for the recombinant EN2 were selected by magnetic-bead SELEX using a single-stranded DNA library containing approximately 3 × 10^14^ molecules. After 12 rounds of selection, an H90 aptamer was obtained, then trimmed to be EBA of 50 mer to ensure that random sequences remained and secondary structure was preserved; EBA had *K*_d_ = 8.26 nM (R^2^ = 0.971) (Table S1; Fig. 2), which indicates high binding affinity to EN2. We also demonstrated the affinity of EBA for human EN2 using fluorescence microscopy of an EN2-expressing cell line, MCF-10A^1^. The result (Fig. S3) showed that EBA had binding affinity for human EN2 as well as recombinant EN2.

### Design and signal amplification of aptamer-mediated HCR

**Figure 3:**
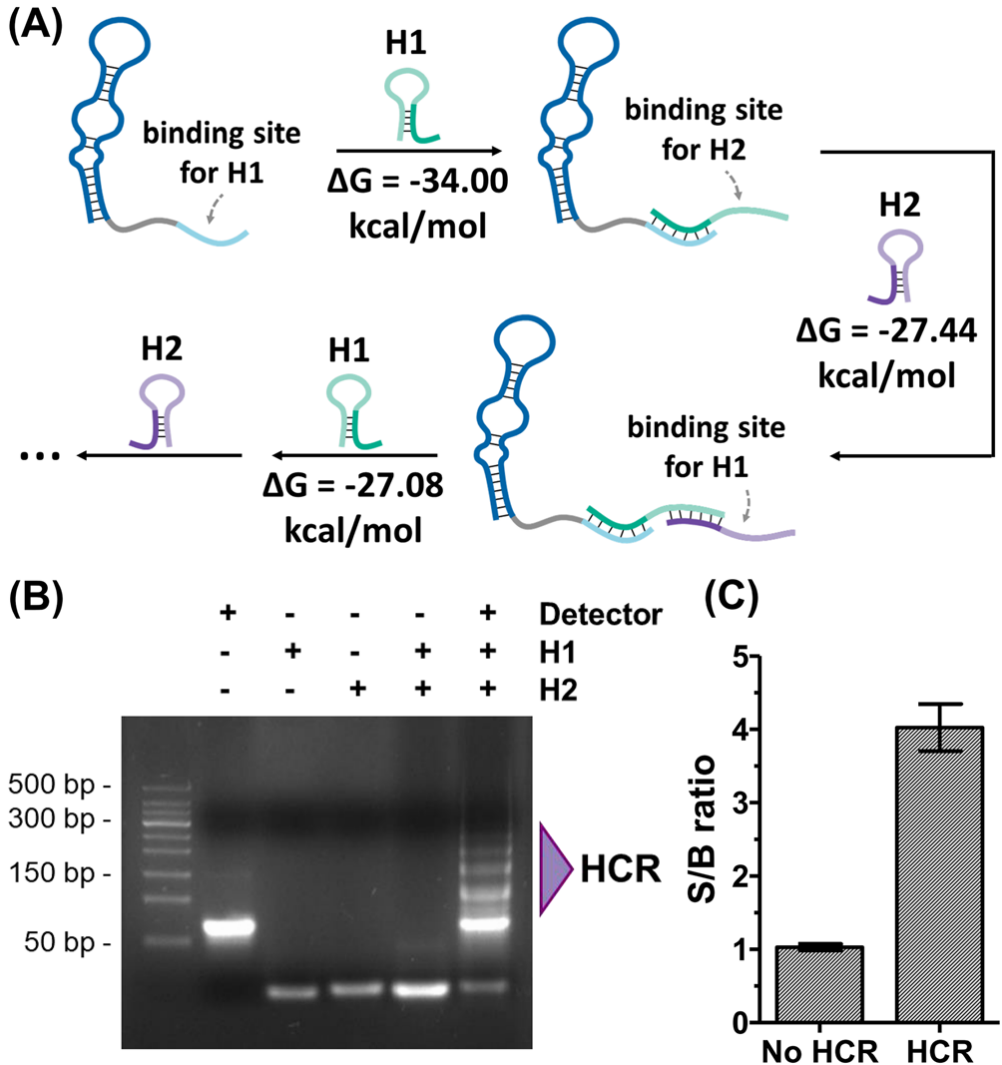
Establishment and effect of aptamer-mediated HCR. (**A**) Schematic description of aptamer-mediated HCR. Calculated Gibbs free energies of hybridization between trigger, H1 and H2 suggested the accessibility of the HCR. (**B**) Confirmation of HCR by gel electrophoresis analysis. Lane 1: T&I 50 bp DNA ladder. Lane 2–4: detector, H1, and H2, respectively. Lane 5: mixture of H1 and H2 (H1 + H2). The hairpins did not hybridize without detector. Land 6: hairpins with detector (H1 + H2 + detector). HCR occurred when all of the reagents existed, with multiple bands representing the HCR products. The original gel is presented in Fig. S4. (**C**) Signal amplification by HCR on the ELONA in response to 10 nM of EN2; the S/B without HCR was 1.03 ± 0.05, whereas the S/B with HCR was 4.00 ± 0.68 (not great, but > 3 ×). Bars: ± s.d., n = 3.

Trigger, H1, and H2 sequence for HCR were designed according to the guideline described in a previous study (Table S2)^21^. To evaluate the HCR feasibility of the designed trigger, H1, and H2 sequences, theoretical calculation was first performed using NUPACK software (www.nupack.org)^22^. The results showed that the Gibbs free energies between trigger and H1 were − 34.00 kcal/mol, and between H1 and H2 were − 27.44 and − 27.08 kcal/mol, respectively, suggesting that HCR is attainable (Fig. 3A).

To test the designed sequences for HCR in experiments, HCR products for the reaction of detector, H1, and H2 were analyzed using gel electrophoresis. Result (Fig. 3B) showed that the designed sequences could generate HCR products only when all of reagents existed in a reaction. Taken together, the results suggest that the HCR products could interact with EN2 and consequently increase the signal-to-background ratio (S/B), which is defined as *A*_EN2_/*A*_blank_, where *A*_EN2_ represents the absorbance at 450 nm in the presence of EN2, and *A*_blank_ represents the absorbance of buffer solution at 450 nm. To compare the S/B, the absorbance intensities of 10 nM EN2 were measured in the ELONA without and with HCR. The S/B without HCR was 1.03 ± 0.05, whereas the S/B with HCR was 4.00 ± 0.68 (Fig. 3C). We also measured signals without the detector to ensure the signal amplification by HCR rather than hairpins H1 and H2 themselves (Fig. S5). Consequently, the HCR increased S/B, and accordingly the sensitivity of the sensor.

### Optimization of the key parameters

To achieve the highest sensitivity of EN2 detection, we optimized the conditions, including antibody concentration, parameters affecting HCR, dilution degree of poly-HRP, and binding time of poly-HRP. Each component was determined using a maximum Δ*A* (*A*_EN2_–*A*_blank_). First, the optimal concentration of antibody as a capture agent was determined to be 5 µg/mL (Fig. S6). Of parameters that affect HCR, the reaction buffer, spacer length between EBA and trigger, and particular order in which related reactions occur were considered, to facilitate both EN2 binding of EBA and HCR of trigger (Fig. S7). Signal intensity was highest when binding buffer was used and 10 consecutive adenosine monophosphates (10A) were inserted as a spacer. There was no difference between pre-HCR and pro-HCR; HCR was performed before (pre-HCR) or after (pro-HCR) the detector bound to EN2. We decided to pre-HCR for further applications because of time efficiency. Among the parameters that affect HCR, reaction buffer had a significant effect on Δ*A*. The binding buffer contained both MgCl_2_ and tween-20, whereas tris buffer previously reported in the literature^15^ contained only MgCl_2_. MgCl_2_ and tween-20 participate in stabilization of DNAs and proteins, respectively^23,24^, and these effects may have improved the capabilities of the detector. In addition, incorporation of 1:500 diluted poly-HRP for 5 min was sufficient to generate signals; 1:250 diluted poly-HRP was not selected because it had a high background signal (Fig. S8). These processes yielded an optimal ELONA for detection of EN2.

### Analytical characteristics of the ELONA

**Figure 4:**
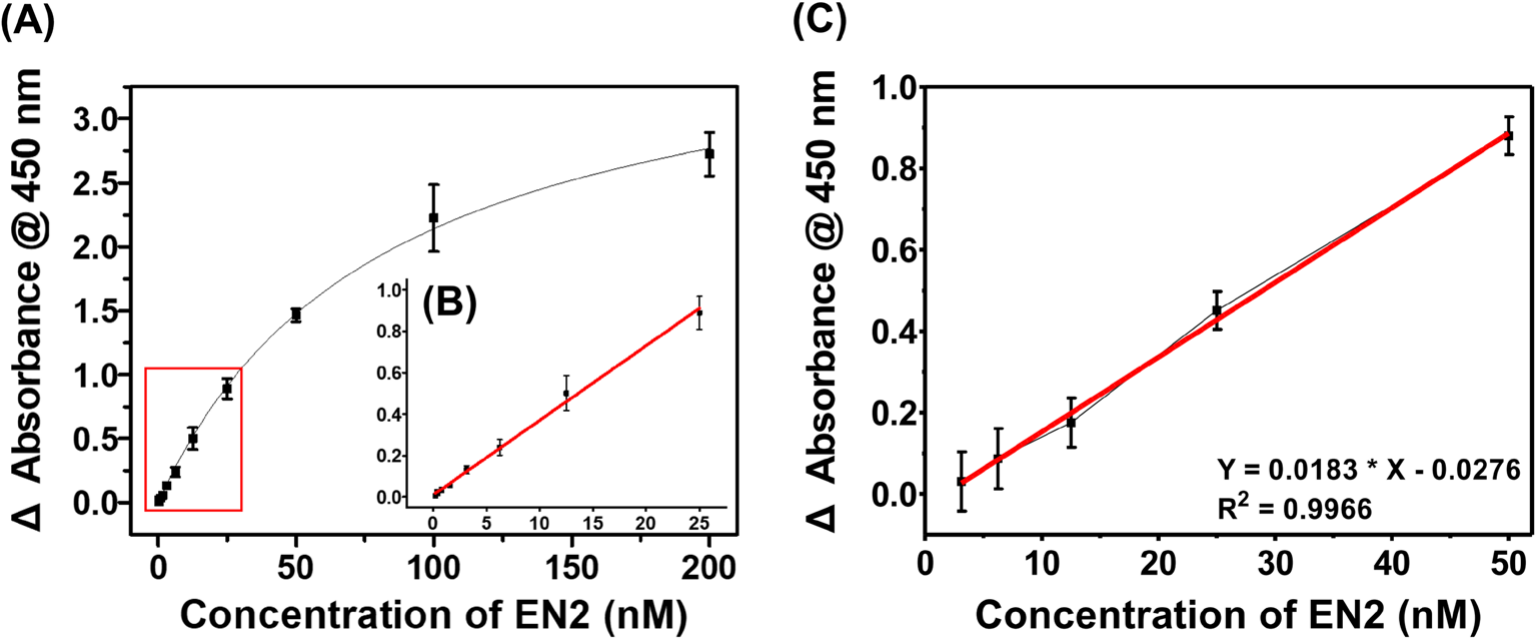
HCR-based aptamer-antibody hybrid ELONA assay for EN2 detection. (**A**), (**B**) ELONA analysis under binding buffer conditions. The calibration curve was obtained in a dynamic range from 0.39 nM to 25 nM, and the LOD was 0.34 nM. (**C**) ELONA analysis for EN2-spiked AUM samples. AUM was diluted in two-fold prior to spiking; the dynamic range was 3.12 nM to 50 nM, with an LOD of 2.69 nM. Bars: ± s.d., n = 3.

Once the reaction conditions were established, we assessed the dynamic range, sensitivity, specificity, and stability of ELONA. We evaluated the dynamic range and sensitivity by measuring absorbance intensities from buffers containing EN2 in the range of 0 nM to 200 nM (Fig. 4A). The ELONA showed a good linear relationship between the concentrations of EN2 and signal intensities (R^2^ = 0.9906), so the dynamic range was 0.39 nM to 25 nM and the limit of detection (LOD) was 0.34 nM (11.6 ng/mL). The average coefficient of variation (CV) was 7.08%, which confirms great reproducibility (Fig. 4B). The data are meaningful in that the mean urine EN2 level is 5.76 nM (197 ng/mL) for bladder cancer patients^2^, and is 23.5 nM (804 ng/mL) with the cut-off value of 1.24 nM (42.4 ng/mL) for prostate cancer patients^7,25^.

We expanded the capability of the ELONA to enable the detection of EN2 in clinical conditions by applying it to artificial urine medium (AUM). Prior to the urine-spiking test, the AUM percentage of the solution for the ELONA was optimized to be 50% (v/v) (Fig. S9); the signal intensities of the EN2-spiked 5% AUM solutions were higher than those of the 50%, but 50% was selected because of the low background signal and low dilution of EN2 concentrations in real clinical samples. In the EN2-spiked AUM samples, the absorbance curves from 0 to 800 nM were obtained (Fig. S10). The dynamic range was determined from 3.12 nM to 50 nM with a LOD of 2.69 nM and R^2^ = 0.9966 (Fig. 4C); the real minimal detectable content of EN2 in urine samples is 5.38 nM (184 ng/mL). The average CV was 13.4%, and the recoveries, calculated as the percentage of the practical concentration divided by the theoretical concentration, varied from 88.8% to 105% (Table S3).

**Figure 5:**
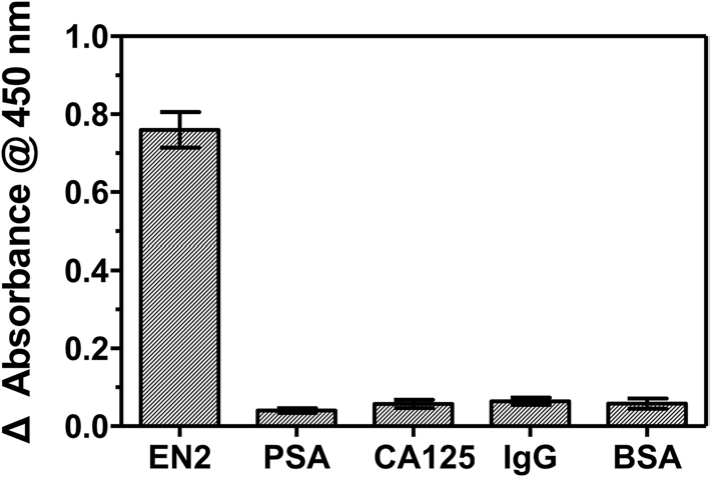
Specificity test with various proteins at 50% AUM. 30 nM of EN2 and 150 nM of other proteins were used for testing. Bars: ± s.d., n = 3.

The specificity of the ELONA was evaluated in AUM using prostate specific antigen (PSA), cancer antigen 125 (CA125), IgG, and bovine serum albumin (BSA) (Fig. 5); PSA and CA125 are known as genitourinary-related tumor markers and can coexist in clinical samples^26,27^, which may interfere with accurate detection of EN2. Signals for proteins other than EN2 were negligible. These results suggested that the developed ELONA is reproducible and reliable to detect EN2 in urine-added conditions.

The ELONA was stable enough to be stored for more 3 months at 4 °C (Fig. S11); this stability permits use of pre-coated plates, and can thereby reduce the detection time to < 2.5 h. Thus, the developed ELONA can be practically applied to the diagnosis of bladder and prostate cancer by testing urine. In order to further highlight the advantages of the designed ELONA in detecting EN2, the analytical properties were compared with those of other methods (Table 1).

**Table 1:** Comparison of the proposed ELONA with conventional bladder or prostate cancer diagnostics and other EN2 detection methods.

Test	Mechanism of detection	Testing material	Detection time	Usability	Performances	References
**Conventional methods for prior screening bladder or prostate cancer**						
Cystoscopy	A thin camera (cystoscope) into the urethra to look inside the bladder	Invasive (−)	Real-time monitoring	Expert required	Variable clinical sensitivity range (68.3–100%) and clinical specificity range (57.0–97.0%)	^28^
DRE	A gloved, lubricated finger into the rectum to check the prostate gland	Invasive (−)	Real-time monitoring	Expert required	Variable clinical sensitivity range (49.0–69.2%) and clinical specificity range (18.0–99.5%)	^34^
PSA test	An immunoassay detecting prostate specific antigen (PSA)	Invasive (serum)	Variable	Accessible	Variable clinical sensitivity range (66.7–100%) and clinical specificity range (18.0–100%)	^34^
**Methods for detecting EN2 in literature**						
LFA	Lateral flow assay (LFA) using anti-EN2 antibody coupled to a colored conjugate	Non-invasive (urine)	20 min	High accessible	No analytical data on performances. Simple and fast method, but requires modified antibodies	^35^
Electrochemical sensor	An electrochemical sensor using aptamer-modified gold electrodes	Non-invasive (urine)	1 day	Less accessible	Ultrasensitive (LOD = 0.0002 ng/mL), but requires at least 16 h electrode fabrication	^36^
Electrochemical sensor	An electrochemical sensor using aptamer-modified carbon-graphene electrodes	Non-invasive (urine)	5.5 h	Less accessible	Insufficient sensitivity (LOD = 1320 ng/mL) and requires 5 h for electrode fabrication	^37^
ELONA	Aptamer-antibody hybrid ELONA	Non-invasive (urine)	2.5 h	Accessible	Sufficient sensitivity (LOD = 11.6 ng/mL), accessibility, low cost, and short turnaround time	This work

## Discussion

All conventional methods to test for bladder or prostate cancer are invasive, and among of them, cystoscopy and digital rectal examination (DRE) involve instrumentation. They have disadvantages such as low sensitivity, a need for highly-skilled specialists, and possibility of inducing injury or infection^28,29^, so they are unsuitable as a routine diagnosis for early detection.

To improve the accessibility of diagnosis, several methods have been developed to detect EN2, a promising urinary biomarker for bladder and prostate cancer. LFA is a highly accessible method because of the simplicity and short detection time. However, it is costly because it uses only of antibodies for recognition and signal transduction, and specifically modifies the antibody by conjugating it with a colored marker. Electrochemical sensors, utilizing aptamers instead of antibodies, are relatively economical, but they need a lot of time and process to fabricate electrodes with pretreatment.

To develop a simple bladder and prostate cancer diagnostic tool for timely monitoring, we discovered a biomarker EN2-binding ssDNA aptamer with a high affinity (*K*_d_ = 8.26 nM), and developed an HCR-based aptamer-antibody hybrid ELONA that directly detects EN2 in urine. In the ELONA, aptamer and anti-EN2 were paired as detector and capture agent, respectively, because the aptamer could be applied to HCR based on the characteristics of nucleic acids for signal amplification. The developed ELONA achieved sensitive detection of EN2 in buffer and AUM, with low LOD of 0.34 nM and 2.69 nM, respectively, and showed specificity by not detecting other urinary proteins. Also, the assay has a short detection time (< 2.5 h) and high stability at 4 °C for at least three months.

ELONA developed here is accessible, because it is a non-invasive method to diagnose bladder or prostate cancer. It is also inexpensive and fast, does not require modified antibodies, has high stability, and numerous samples can be tested simultaneously. Therefore, it is sufficient to replace the conventional methods to diagnose bladder or prostate cancer as regular screening, and may facilitate timely treatment and reduction of disease-specific mortality. This study is the first to develop ELONA for EN2 in urine.

## Materials and methods

### Materials

Genomic DNA of EN2 was purchased from Thermo Fisher Scientific (USA). The pET28a plasmid was purchased from Novagen (Germany). Nucleospin Gel and PCR Clean-up/Plasmid EasyPure kit were acquired from Macherey–Nagel (Germany). *E. coli* strain Rosetta (DEC), Dynabeads his-tag Isolation and Pulldown, MyOne Streptavidin C1, and TOPO TA cloning kit were purchased from Invitrogen (USA). HisTrap Ni–NTA affinity column and Sephadex G-25 Superfine resin were purchased from GE Healthcare (USA). All DNAs were synthesized from Bionics Inc. (South Korea). For ELONA, EN2 monoclonal antibody was purchased from Abnova (Taiwan). Streptavidin Poly-HRP (poly-HRP), 3,3′,5,5′-tetramethylbenzidine (TMB) substrate kit, 96-well polystyrene plates, and sulfuric acid (H_2_SO_4_) were purchased form Thermo Fisher Scientific (USA). Bovine serum albumin (BSA) and IgG were purchased from Sigma-Aldrich (USA). Prostate specific antigen (PSA) and cancer antigen 125 (CA125) were purchased from Sino Biological Inc. (USA) and Abnova (Taiwan). Artificial urine medium (AUM) was purchased form Pickering Laboratories Inc. (USA). All materials were of analytical grade. All aqueous solutions were prepared in deionized water (> 18 MΩ) obtained using a Direct-Q system from Merck Millipore (USA).

### Preparation and purification of EN2

The EN2 genes, which corresponds to protein P19622 (UniProtKB database), were amplified using PCR with sequence specific-forward primers including BamHI linker (EFP, 5’-CCC GGA TCC ATG GAG GAG AAT GAC CCC AAG C-3’) and reverse primers including XhoI linker (ERP, 5’-CCC CTC GAG CTA CTC GCT GTC CGA CTT GC-3’). PCR products were purified, and subsequently digested using BamHI and XhoI. They were cloned into pET28a vectors that had been pre-digested under the same conditions, which leads to the addition of the His-tag at the N-terminal of EN2. The plasmids were then transformed into *E. coli* Rosetta (DEC) to allow over-expression of EN2.

Bacteria were grown at 37 °C in lysogeny broth (LB) supplemented with 50 µg/mL kanamycin and 25 µg/mL chloramphenicol until the optical density (OD) at 600 nm reached 0.6. Point expression was induced by addition of 200 μM isopropyl β-d-1-thiogalactopyranoside (IPTG) and incubation at 37 °C for an additional 6 h.

Bacterial cells were harvested and sonicated, then the lysate was cleared by centrifugation at 18,000 rpm for 40 min and applied to a HisTrap Ni–NTA column. The fusion proteins were eluted with an imidazole gradient, then the eluates were added to a desalting column with storage buffer (50 mM Tris–HCl, 100 mM NaCl, 0.5 mM β-mercaptoethanol, and 5% (v/v) glycerol, pH 8.0). The products were stored at − 80 °C in aliquots containing 20% (v/v) glycerol, then analyzed using 12.5% sodium dodecyl sulfate–polyacrylamide gel electrophoresis (SDS-PAGE).

### In vitro selection for EN2-specific ssDNA aptamer

The overall in vitro selection was conducted using magnetic bead SELEX and performed in 100 μL of binding buffer (20 mM Tris–HCl, 50 mM NaCl, 5 mM KCl, 5 mM MgCl_2_, pH 8.0)^30,31^. A library template was synthesized as ssDNA containing a central random region of 40 nucleotides (DNA library: 5’-CAC CTA ATA CGA CTC ACT ATA GCG GAT CCG A-N_40_-CTG GCT CGA ACA AGC TTG C-3’). For the selection, 20 μL of pre-washed NTA magnetic beads (Dynabeads His-Tag Isolation & Pulldown) were incubated with 500 pmol of His-tagged EN2 for 1 h at room temperature (RT), then washed to remove unbound proteins using an external magnetic separator. Then 500 pmol of ssDNA library was heated at 95 °C for 5 min then cooled on ice for 1 h to stabilize the naturally-occurring secondary structures, then incubated with EN2-immobilized magnetic beads for 1 h at RT. The incubation time of protein and ssDNAs was gradually decreased from 1 h to 30 min as the rounds progressed. Unbound ssDNAs were collected and measured by UV absorbance at 260 nm to calculate the amount of bound ssDNAs.

Then the EN2-ssDNA complexes were eluted using binding buffer supplemented with 300 mM imidazole, and the eluted ssDNAs were precipitated using 70% (v/v) ethanol and amplified by PCR with *pfu* polymerase, using forward primers (5’-CAC CTA ATA CGA CTC ACT ATA GCG GA-3’) and biotinylated reverse primers (5’-biotin-GCA AGC TTG TTC GAG CCA G-3’). The resulting dsDNAs were added to 70 μL of streptavidin-coated magnetic beads (Dynabeads MyOne Streptavidin C1) in coupling buffer (5 mM Tris–HCl, 1 M NaCl, 0.5 mM EDTA, 0.0025% (v/v) tween-20, pH 7.5) for 1 h at RT, then washed in coupling buffer. Then non-biotinylated ssDNAs were eluted using 200 mM NaOH and sequentially precipitated using 70% (v/v) ethanol. The generated ssDNAs were used as the library for the next round of SELEX. After the 12th round of SELEX, the eluted ssDNAs from the EN2-immobilized magnetic beads were amplified by PCR using unmodified primers.

Finally, the amplified dsDNAs were cloned into pCR 2.1-TOPO TA vectors, and the constructs were transformed to *E. coli* TOP10 cells (TOPO TA Cloning Kit). The plasmids were purified using a Nucleospin Plasmid EasyPure kit, and the inserts were sequenced. The secondary structures of the aptamer candidates were predicted using the Mfold program (http://www.unafold.org/mfold/applications/dna-folding-form.php)^32^.

### Measurement of dissociation constant (***K***_***d***_)

The dissociation constants *K*_d_ were determined using a magnetic-bead fluorescent assay. 5’- FAM modified aptamers were heated at 95 °C for 5 min then cooled on ice for 1 h in binding buffer prior to use. First, 100 pmol of His-tagged EN2 was shake-incubated with 10 μL of pre-washed NTA magnetic beads for 1 h at RT, then the EN2-immobilized beads were separated using an external magnetic separator. Next, the EN2-immobilized beads were reacted with several concentrations (0–100 nM) of 5’-FAM modified aptamers for 1 h at RT in darkness. The suspensions were washed twice to remove unbound aptamers, then the EN2-aptamer complexes were obtained using binding buffer supplemented with 300 mM imidazole. Finally, the fluorescence intensities of the eluates were measured by 485/20 nm excitation and 528/20 nm emission filter on a Synergy™ HT microplate reader (BioTek, USA). *K*_d_ values were calculated by fitting the data to the one-site-binding (total and nonspecific binding) model of GraphPad Prism 5.0 software (GraphPad, USA)^33^.

### Aptamer-antibody hybrid ELONA that uses HCR

In preparation, 5’-biotinylated detector, -hairpin 1 (H1), and -hairpin 2 (H2) were heated at 95 °C for 5 min, then cooled on ice for 30 min in binding buffer. First, 96-well polystyrene plates were coated with 5 μg/mL of EN2 monoclonal antibody (100 μL) at 4 °C overnight. The plates were blocked using 200 μL of blocking buffer (10 mg/mL BSA in PBS) for 1 h at RT. Subsequently, the plates were washed with wash buffer (0.05% (v/v) tween-20 in PBS), then a two-fold serial dilution of EN2 (0, 0.20, 0.39, 0.78, 1.56, 3.13, 6.25, 12.5, 25, 50, 100 and 200 nM) in PBS was added individually to wells of the plates for 1 h at RT. At the same time, detector, H1, and H2 were mixed to 100 nM each, then reacted for 1 h at RT to complete the HCR process. Then the plates were washed three times with wash buffer, and the HCR products were treated for 1 h at RT. The plates were washed three times, then incubated with 100 μL of poly-HRP diluted 1:500 in blocking buffer for 5 min at RT. The plates were washed five times, then reacted with TMB solution for 20 min at RT in darkness. The reactions were stopped by 1 M sulfuric acid, and the absorbance at 450 nm (*A*_450_) was measured using a Synergy™ HT microplate reader (BioTek, USA). For EN2-spiked urine samples, a two-fold serial dilution of EN2 from 800 nM was prepared in PBS including 50% (v/v) AUM, and other procedures were performed as above. All data were obtained in triplicate at each EN2 concentration. To analyze the sensitivity of the assay, the limit of detection (LOD) was calculated as 3*σ/s*, where *σ* is the standard deviation (s.d.) of the sample absorbance, and *s* is the slope of the linear relationship between *A*_450_ and EN2 concentration. The coefficient of variation (CV) was calculated as the ratio of s.d. to the mean.

## Supplementary Information

Supplementary Information.

## Data Availability

The protein sequences used during the study are available in the UniProtKB, P19622.
