# Supplementary material for: Aptamer-antibody hybrid ELONA that uses hybridization chain reaction to detect a urinary biomarker EN2 for bladder and prostate cancer
Source: Sci Rep. 2022 Jul 7;12:11523. doi: 10.1038/s41598-022-15556-1 (PMC9263169; doi:10.1038/s41598-022-15556-1)
Supplement: Supplementary file 1 — Supplementary Information. [file 41598_2022_15556_MOESM1_ESM.pdf]

Supplementary data

Title

Aptamer-antibody hybrid ELONA that uses hybridization chain reaction to detect a urinary biomarker EN2 for bladder and prostate cancer

Authors

Eunseon Kim[1], Minji Kang[1], Changill Ban*[1]

Affiliations

1. Department of Chemistry, Pohang University of Science and Technology, 77, Cheongam-Ro, Nam-Gu, Pohang, Gyeongbuk, 790-784, Republic of Korea

* To whom correspondence should be addressed:

C. Ban: E-mail: ciban@postech.ac.kr; Tel: 82-54-279-2127; Fax: 82-54-279-8649

E-mail addresses of authors:

Eunseon Kim: eunseonkim@postech.ac.kr (E. Kim), Minji Kang: minjikang@postech.ac.kr (M. Kang).

Table of Contents

1 Supplementary Tables and Figures

1.1 Tables S1-S3

1.2 Figures S1-S11

2 Reference

1 Supplementary Tables and Figures

1.1 Tables S1-S3

Aptamer	Sequence (from 5’ to 3’)	Length (nt)
**H90**	5’ – CAC CTA ATA CGA CTC ACT ATA GCG GAT CCG AGG TGA GGA GCG CCG GTG AGA CTC CTT CAT TCT ATC TGC TCC TGG CTC GAA CAA GCT TGC – 3’	90
**EBA (trimmed H90)**	5’ – AGC GGA TCC GAG GTG AGG AGC GCC GGT GAG ACT CCT TCA TTC TAT CTG CT – 3’	50

Table S1. The aptamer sequences discovered by SELEX for EN2. The underlined sequence indicates the EBA.

Name	Sequence (from 5’ to 3’)	Length (nt)
**EBA_A0_trigger**	5’ – AGC GGA TCC GAG GTG AGG AGC GCC GGT GAG ACT CCT TCA TTC TAT CTG CTT TAG GTA ACC GAA TCG CC – 3’	68
**EBA_A5_trigger**	5’ – AGC GGA TCC GAG GTG AGG AGC GCC GGT GAG ACT CCT TCA TTC TAT CTG CTA AAA ATT AGG TAA CCG AAT CGC C – 3’	73
**EBA_A10_trigger (detector)**	5’ – AGC GGA TCC GAG GTG AGG AGC GCC GGT GAG ACT CCT TCA TTC TAT CTG CTA AAA AAA AAA TTA GGT AAC CGA ATC GCC – 3’	78
**EBA_A15_trigger**	5’ – AGC GGA TCC GAG GTG AGG AGC GCC GGT GAG ACT CCT TCA TTC TAT CTG CTA AAA AAA AAA AAA AAT TAG GTA ACC GAA TCG CC – 3’	83
**EBA_A20_trigger**	5’ – AGC GGA TCC GAG GTG AGG AGC GCC GGT GAG ACT CCT TCA TTC TAT CTG CTA AAA AAA AAA AAA AAA AAA ATT AGG TAA CCG AAT CGC C – 3’	88
**Hairpin 1 (H1)**	5’ – AAC CGA ATC GCC TCT CAT GGC GAT TCG GTT ACC TAA – 3’	36
**Hairpin 2 (H2)**	5’ – ATG AGA GGC GAT TCG GTT TTA GGT AAC CGA ATC GCC – 3’	36

Table S2. The sequences for aptamer-mediated HCR. The underlined sequences indicate the sequences served as spacer between aptamer and trigger. The highlighted yellow sequences represent trigger sequences.

Spiked EN2 Concentration (nM)	Measured EN2 Concentration (nM)	Recovery (%)
3.13	3.20	103
6.25	6.27	100
12.5	11.1	88.8
25.0	26.2	105
50.0	49.6	99.3

Table S3. Recoveries of the ELONA in EN2-spiked AUM samples.

1.2 Figures S1-S11

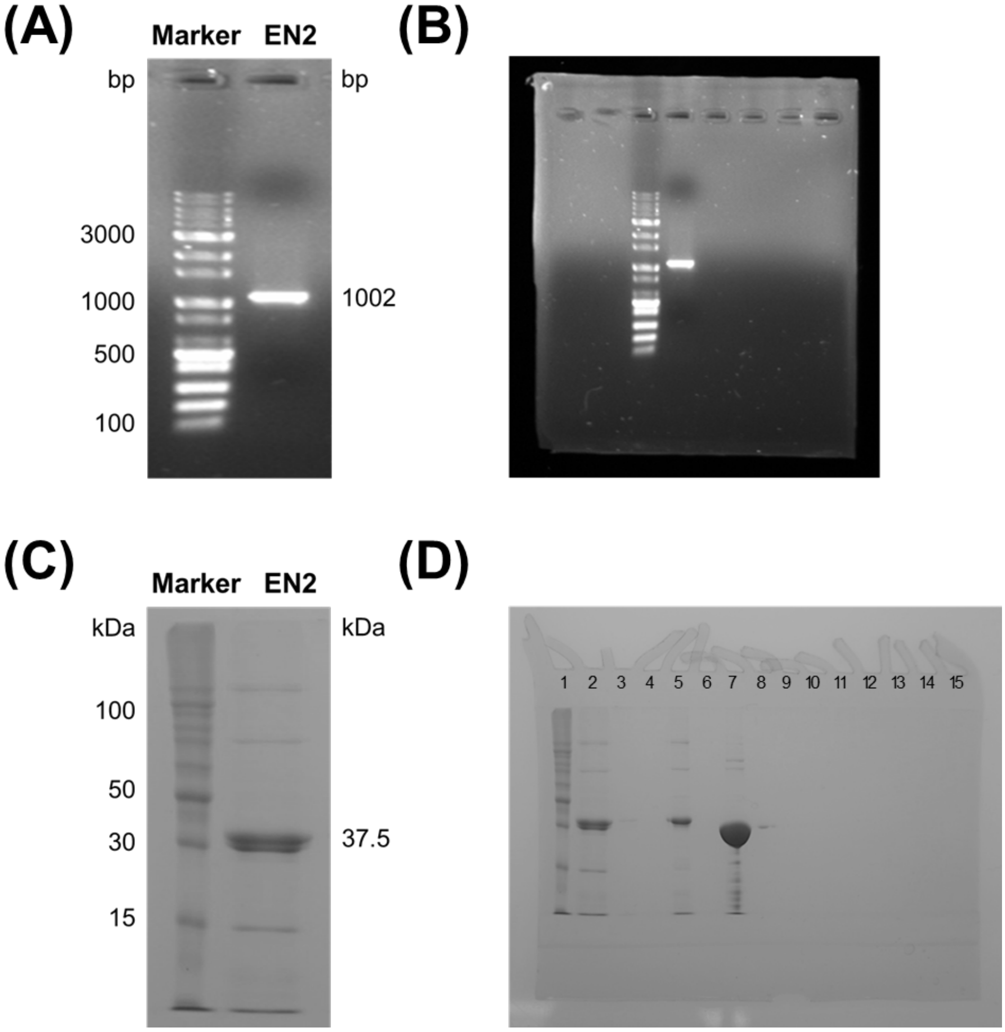

Figure S1. Gel electrophoresis of the recombinant EN2 gene and protein. (A) The PCR product (1002 bp) of EN2 gene at 1% agarose gel in 0.5X TBE buffer, and (B) its original image. Marker is BioFact 1 kb Plus DNA ladder. (C) The purified recombinant EN2 protein (37.5 kDa) at 12.5% SDS-PAGE gel. Marker is Step-view 10 kD Marker. (D) The original image of (C), and Lane 5 and lane 7 are the dilution and concentrate of the solution used in lane 2, respectively.

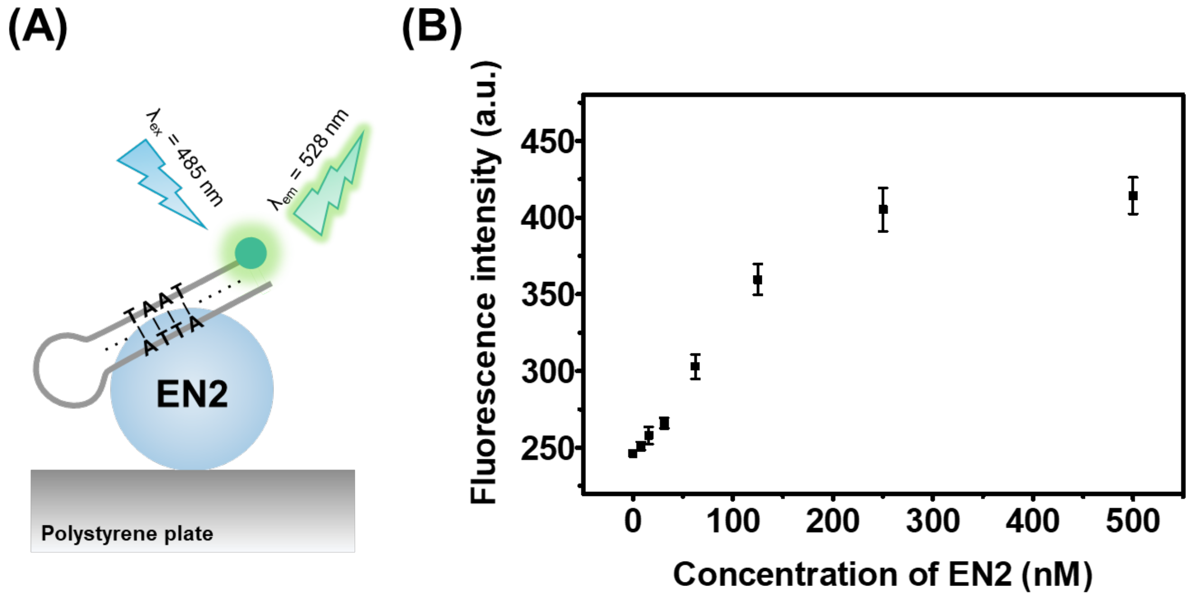

Figure S2. Biological activity of the recombinant EN2. (A) Schematic of the activity assay. The EN2 was directly immobilized on the plate surface. 5' FAM-modified DNA, forming a double-stranded EN2 binding motif (5'-TAAT-3')[1,2], was added, and the EN2 activity was detected by measuring the fluorescence intensities of FAM (485 nm for excitation and 528 nm for emission). (B) Fluorescence intensities for various EN2 concentrations. The signal increased with EN2 concentration and reached a saturated signal at 250 nM. The recombinant EN2 could bind the motif, indicating a conserved activity of EN2 when expressed in bacterial systems. The full sequence of the 5' FAM-modified DNA is as follows: 5'- CGC ATA ATT ACC TCC AGA AGG AGA GGT AAT TAT GCG-3'. Bars: ± s.d., n = 3.

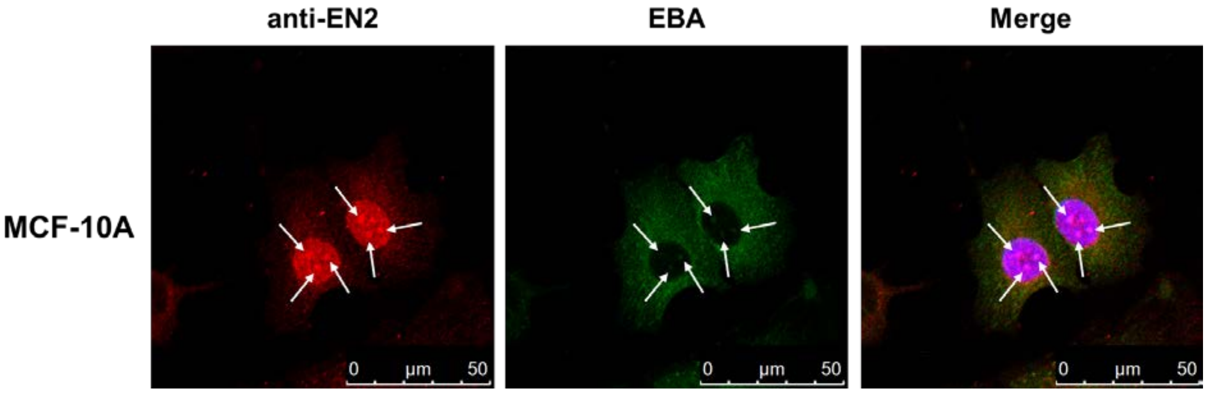

Figure S3. Representative image of EN2 proteins in fixed MCF-10A cells. MCF-10A cell line was purchased from American Type Culture Collection (ATCC). Dulbecco's Modified Eagle's Medium (DMEM), fetal bovine serum (FBS), penicillin and streptomycin were purchased from Hyclone (USA). The cells were maintained in DMEM supplemented with 10% (v/v) fetal bovine serum (FBS), 100 units/mL penicillin and 100 µg/mL streptomycin at 37 °C in 5% CO_2, humidified-cell culture incubator. The cells were passaged when they reached approximately 80% confluence. For cell microscopy, 3×10^5 cells were seeded on a 60 × 15 mm tissue culture dish. Cells were fixed with 4% (v/v) paraformaldehyde (PFA) for 10 min, permeabilized with 0.1% (v/v) Triton X-100 for 10 min and blocked with cell blocking buffer (20 mg/mL BSA in DPBS) for 1h at RT. Then, cells were incubated with anti-EN2 (1:200) for 1 h at 37 °C, followed by staining with the goat anti-mouse Alexa Fluor Plus 555 (1:1000; Invitrogen) in staining buffer (1 mg/mL BSA in DPBS) for 45 min at RT. After washing three times, the cells were stained with 5' FAM-modified EBA (200 nM) in staining buffer for 1h at RT. Finally, they were reacted with 100 µg/mL RNase A, and then with Hoechst 33342 (Invitrogen) for 10 min at RT to stain the nucleus. EN2 protein signal was captured using a Leica TCS SP5 laser scanner (Leica Microsystems, Germany) and the images were processed using LAS AF Lite. The arrows indicate overlapping signals between anti-EN2 and EBA; showing the affinity of EBA for human EN2. Scale bar = 50 µm.

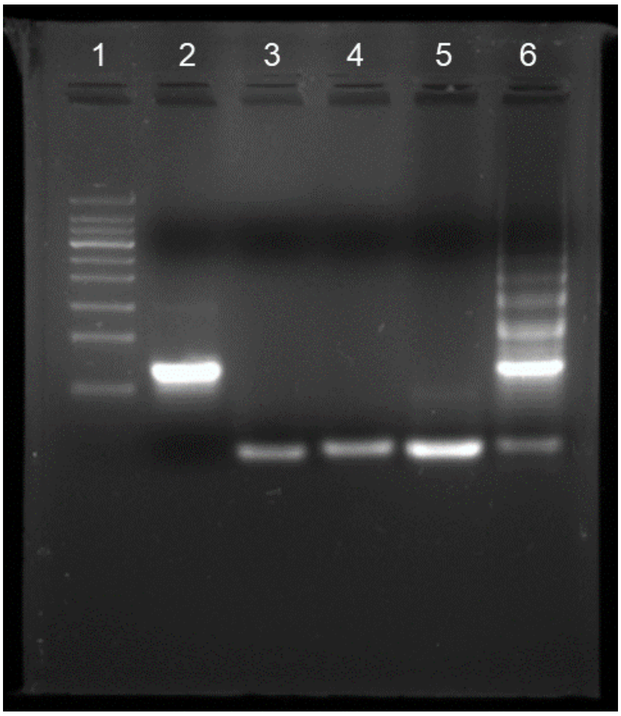

Figure S4. Uncropped gel image of Fig. 3B. Lane information: first lane is T&I 50 bp DNA ladder. Lane 2 is detector, lane 3 is H1, lane 4 is H2, and lane 5 is the mixture of H1 and H2. Lane 6 is the HCR products; the detector was incubated with H1 and H2 with a final concentration of 3 uM at RT for 1 h, and then analyzed on a 3% agarose gel.

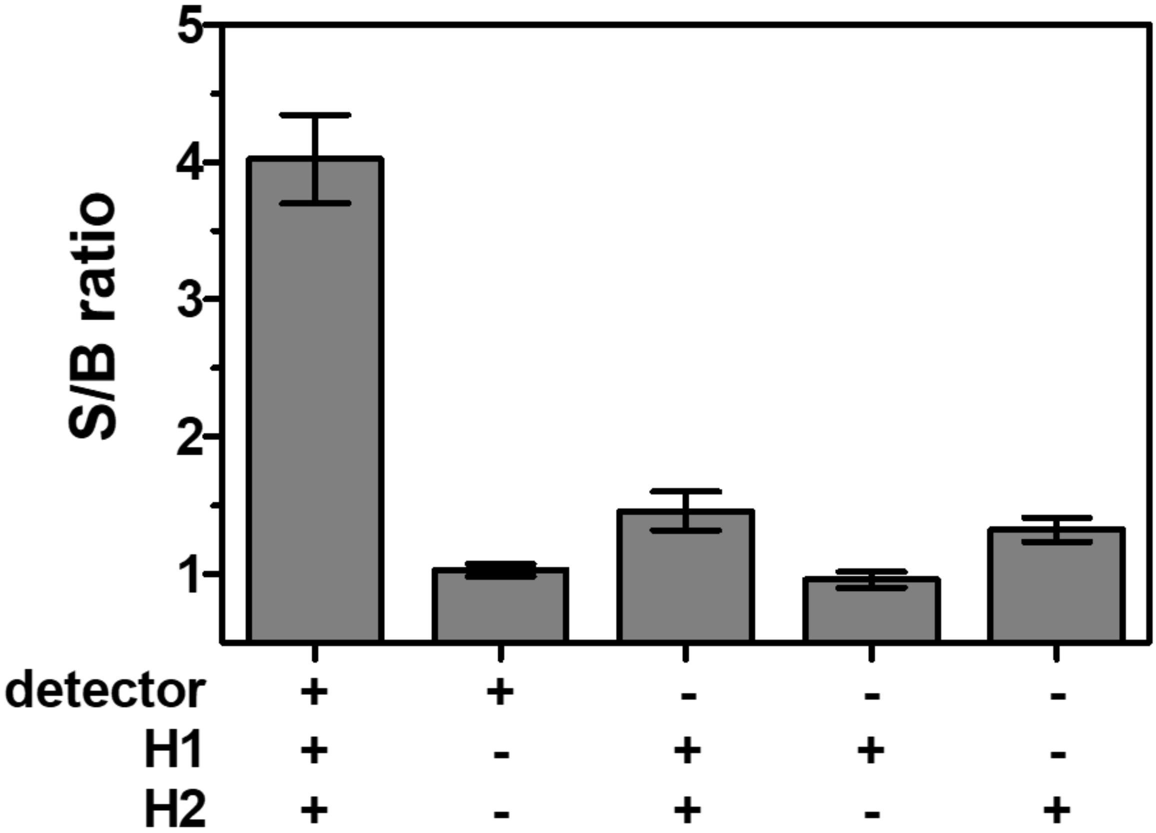

Figure S5. Signal amplification by HCR. Signal was meaningful when all components of the HCR were present, meaning that signal amplification occurred only in the HCR not in the hairpins H1 and H2. Bars: ± s.d., n = 3.

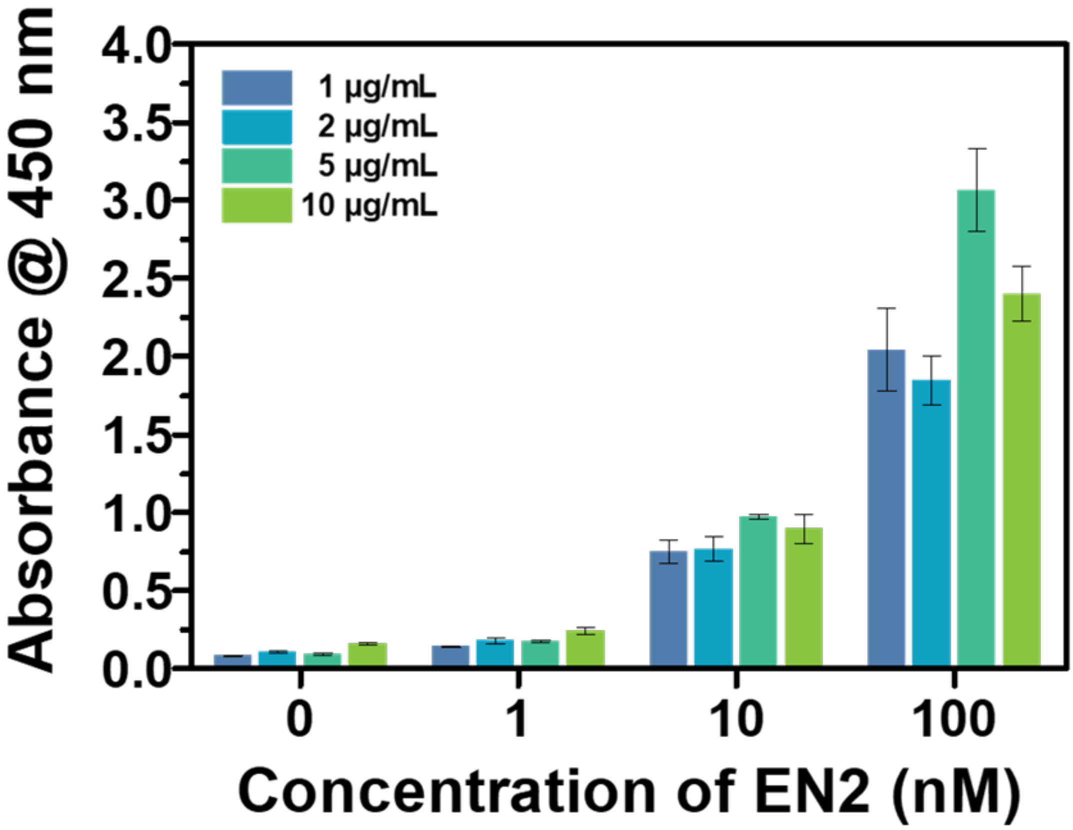

Figure S6. Optimization of EN2 antibody concentration. The optimal antibody concentration was determined by treating 0-100 nM of EN2 protein with various concentrations of EN2 antibody (1, 2, 5 and 10 ug/mL) in the ELONA. Bars: ± s.d., n = 3.

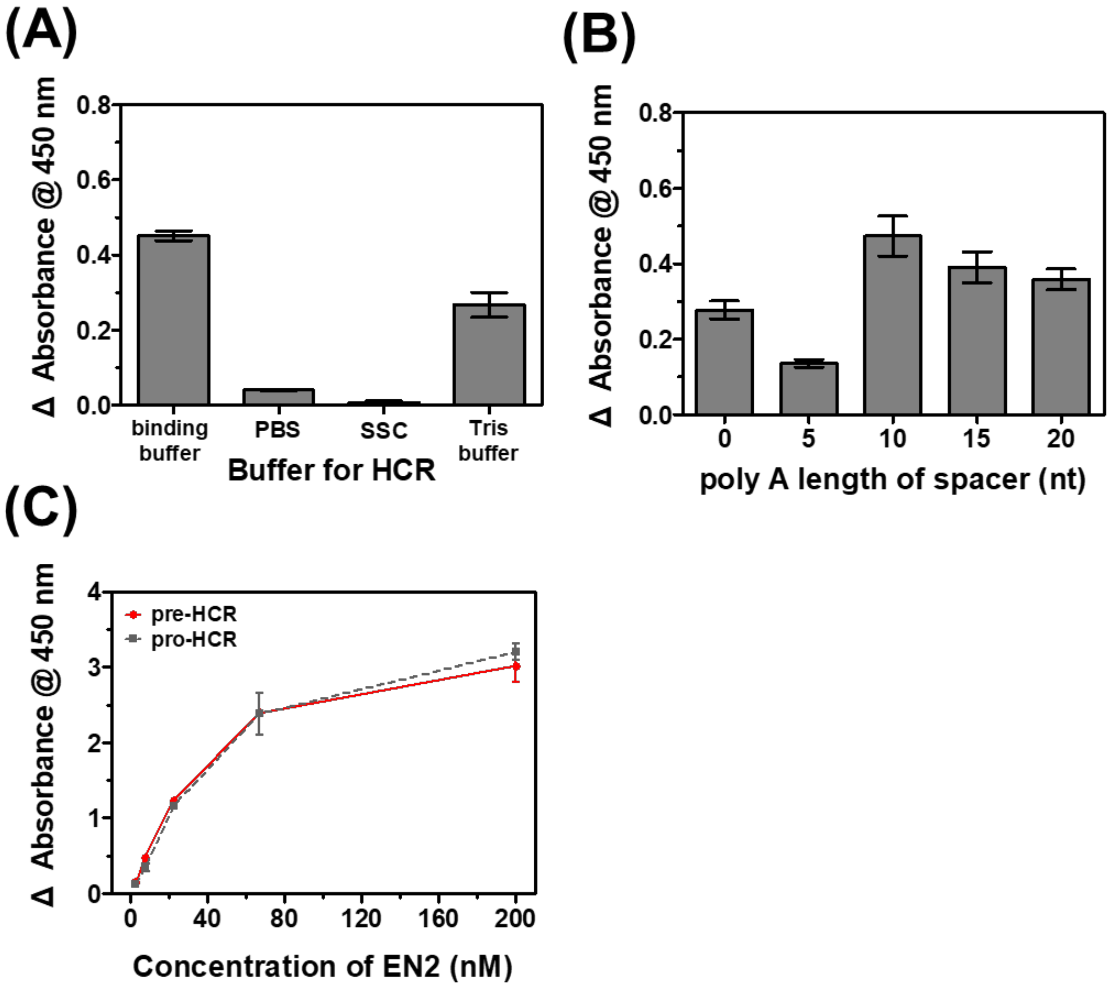

Figure S7. Optimization of HCR-related key parameters for EN2 detection. (A) The effect of HCR buffer. HCR was conducted in four kinds of buffers; binding buffer, PBS, SSC (150 mM $NaCl_2$, 15 mM $Na_3C_6H_5O_7$, pH 7.0), and tris buffer[3] (20 mM Tris-HCl, 300 mM NaCl, 5 mM $MgCl_2$, pH 7.6). (B) Various lengths of spacer. 0 – 20 additional adenosine monophosphates (0A – 20A) were inserted as a spacer between EBA and trigger. (C) The operation order of HCR. HCR was performed before (pre-HCR) or after (pro-HCR) the detector bound to EN2. In each process, the other parameters were fixed at the optimized conditions (binding buffer, 10A, and pre-HCR). Bars: ± s.d., n = 3.

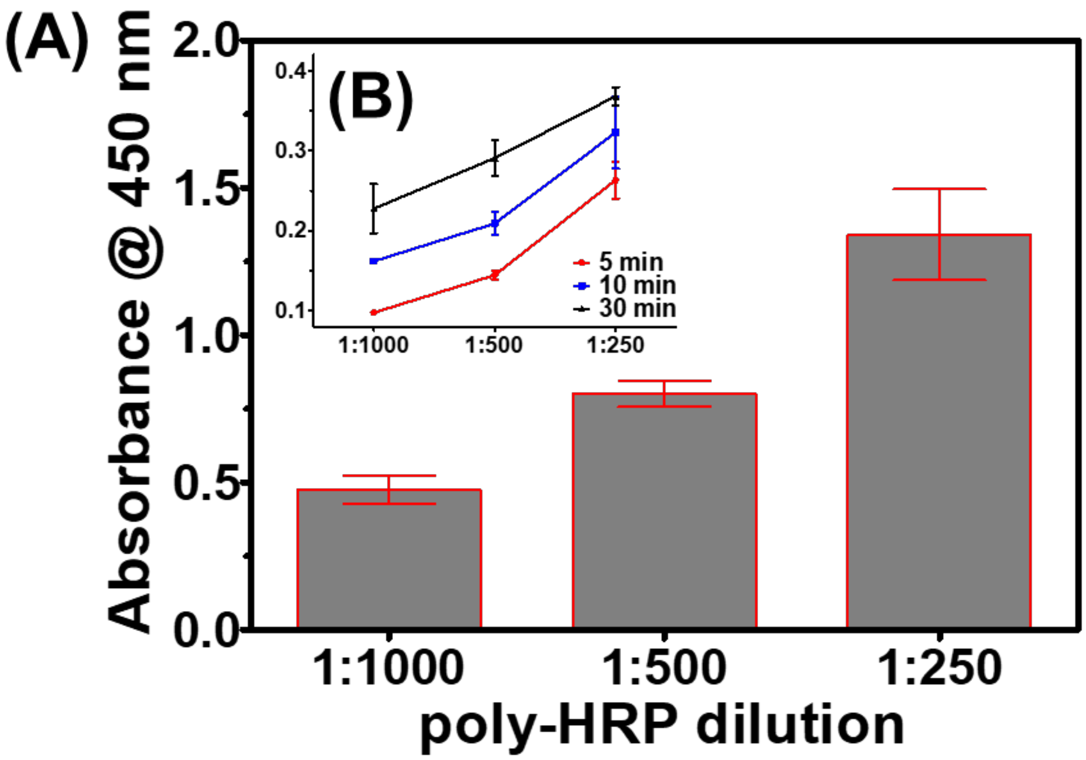

Figure S8. Optimization of poly-HRP related parameters. (A) Degree of poly-HRP dilution. 10 nM EN2 was detected by the ELONA with three dilution ranges (1:1000, 1:500, and 1:250) of poly-HRP. The binding time of poly-HRP was 5 minutes. (B) Background signals upon detection of EN2 with various dilution ranges and binding times of poly-HRP. The less the dilution and the longer the binding time, the stronger the background signal.

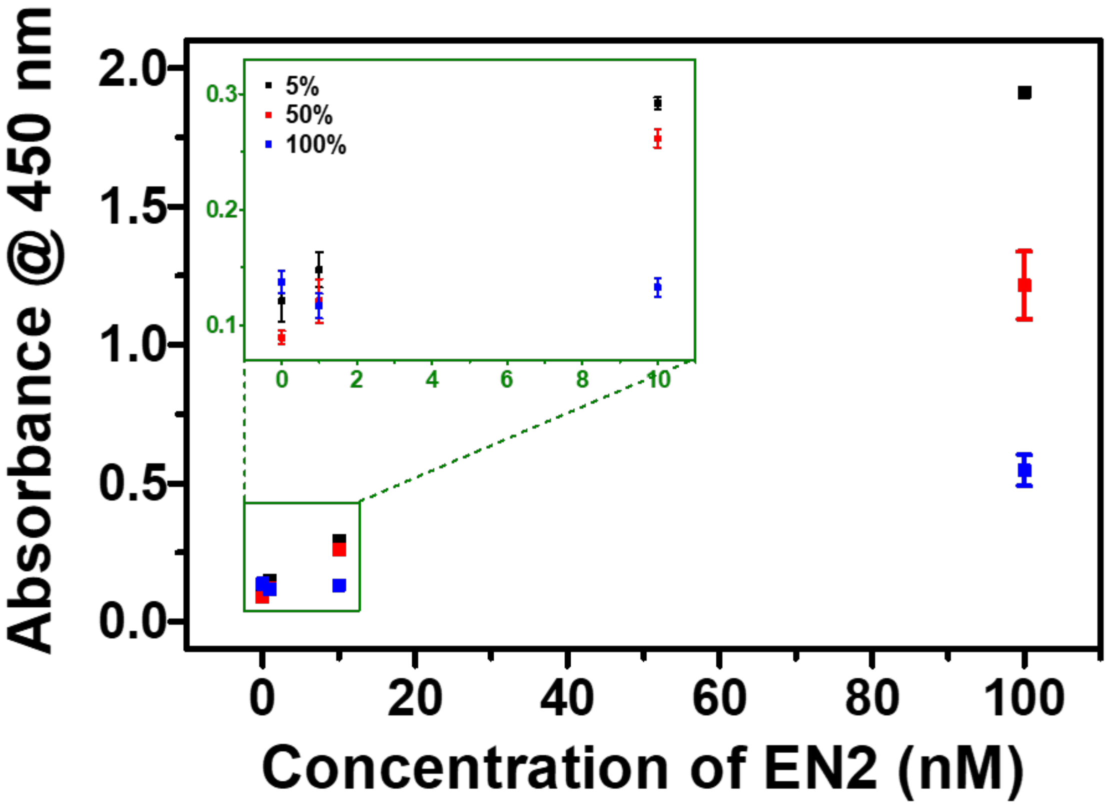

Figure S9. Optimization of AUM percentage for clinical applications. EN2 concentrations (0, 1, 10, and 100 nM) were detected in the ELONA with various buffer conditions; PBS buffer contacting 5%, 50%, and 100% (v/v) AUM. Bars: ± s.d., n = 3.

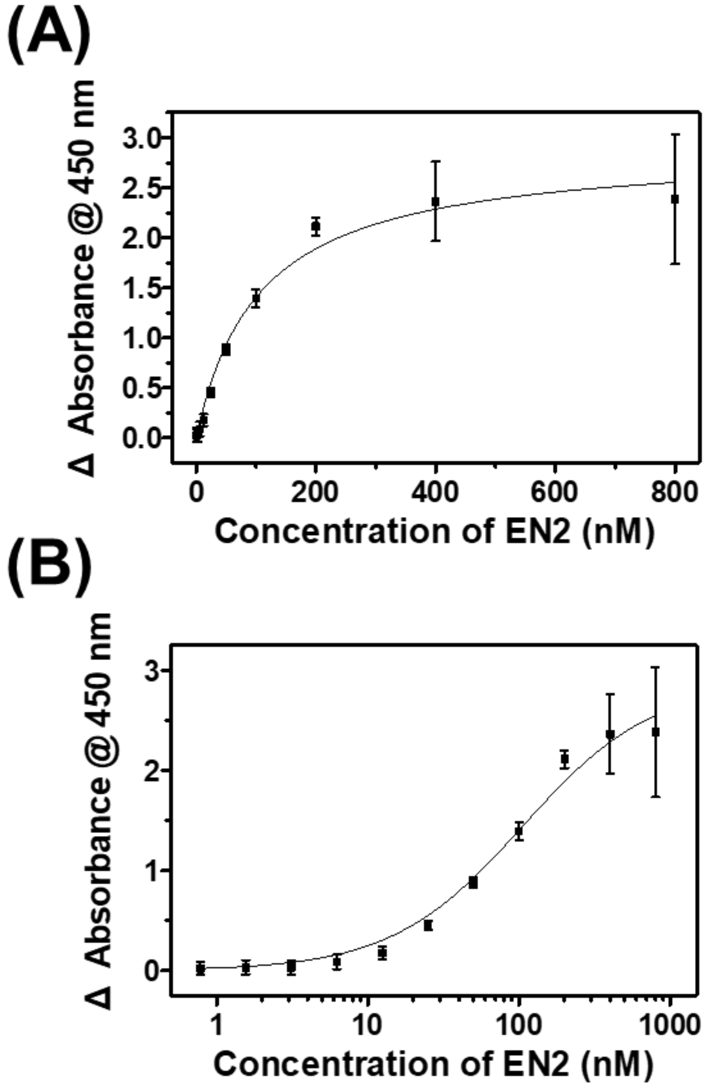

Figure S10. ELONA assay for two-fold serial dilutions of EN2 ranging from 0.78 nM to 800 nM in AUM conditions. (A) Calibration curve, and (B) S-curve of the analysis. Bars: ± s.d., n = 3.

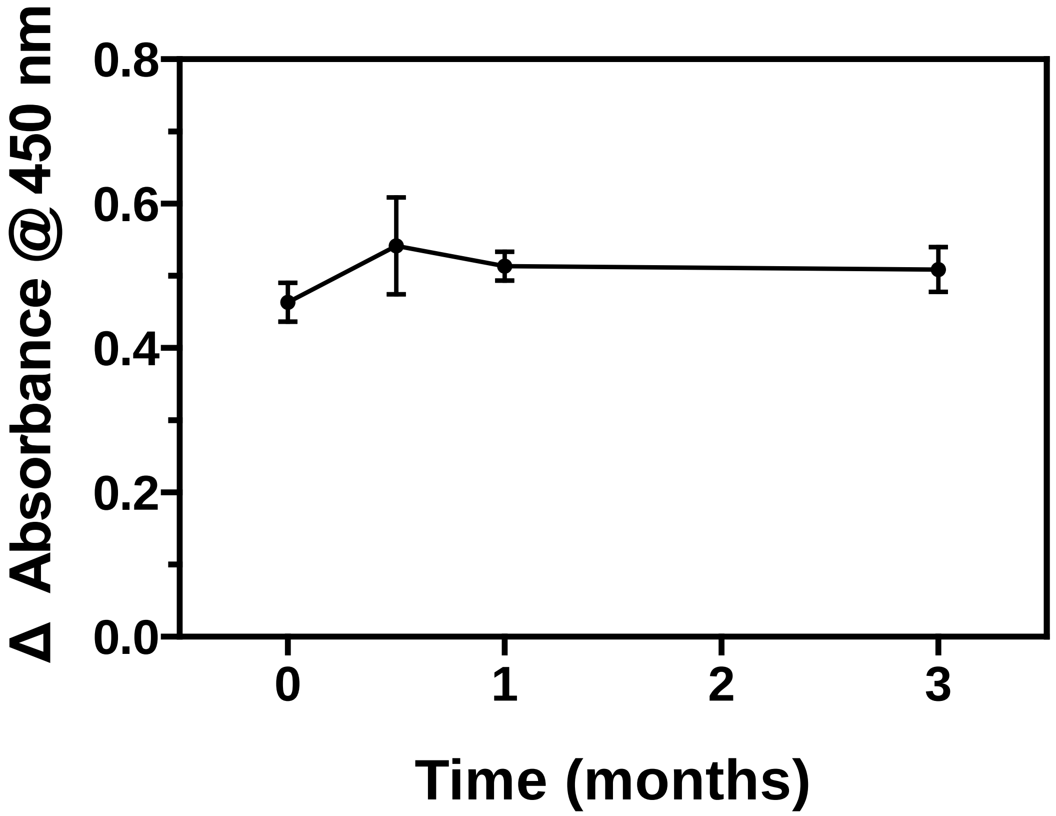

Figure S11. Long-term stability of the ELONA. After the EN2 antibody attachment and blocking processes, the plates were dried and stored at refrigerator until 3 months. HCR products were also stored together. Signals of 10 nM EN2 were maintained for at least 3 months, supporting the usability of the ELONA.

2 Reference

1 Gehring, W. J., Affolter, M. & Bürglin, T. HOMEODOMAIN PROTEINS. *Annual Review of Biochemistry* **63**, 487-526, doi:10.1146/annurev.bi.63.070194.002415 (1994).

2 Kissinger, C. R., Liu, B., Martin-Blanco, E., Kornberg, T. B. & Pabo, C. O. Crystal structure of an engrailed homeodomain-DNA complex at 2.8 Å resolution: a framework for understanding homeodomain-DNA interactions. *Cell* **63**, 579-590 (1990).

3 Dirks, R. M. & Pierce, N. A. Triggered amplification by hybridization chain reaction. *Proceedings of the National Academy of Sciences* **101**, 15275-15278 (2004).
